# Pluripotency‐associated genes reposition during early embryonic developmental stages in pigs

**DOI:** 10.1111/asj.13408

**Published:** 2020-06-23

**Authors:** Hiep Thi Nguyen, Nguyen Trong Nghia, Nghiem Thi Ha Lien, Thanh Quang Dang‐Nguyen, Nguyen Thi Men, Nguyen Viet Linh, Bui Xuan Nguyen, Junko Noguchi, Hiroyuki Kaneko, Kazuhiro Kikuchi

**Affiliations:** ^1^ Institute of Agrobiological Sciences National Agriculture and Food Research Organization (NARO) Tsukuba Japan; ^2^ The United Graduate School of Veterinary Science Yamaguchi University Yamaguchi Japan; ^3^ Institute of Biotechnology Vietnam Academy of Science and Technology Hanoi Vietnam; ^4^ Institute of Physics Vietnam Academy of Science and Technology Hanoi Vietnam

**Keywords:** allelic expression, gene positioning, pluripotency‐associated genes, porcine embryos

## Abstract

We examined the allelic expression and positioning of two pluripotency‐associated genes, *OCT4* and *SOX2*, and two housekeeping genes, *ACTB* and *TUBA*, in 4‐ and 8‐cell porcine embryos utilizing RNA and DNA fluorescence in situ hybridization (FISH) in single blastomeres. The proportion of blastomeres expressing *SOX2* bi‐allelically increased from 45% at the 4‐cell stage to 60% at the 8‐cell stage. Moreover, in 8‐cell embryos, *SOX2* was expressed bi‐allelically in significantly more blastomeres than was the case for *OCT4*, and this was associated with a tendency for *SOX2* alleles to move toward the nuclear interior during 4‐ to 8‐cell transition. However, the radial location of *OCT4* alleles did not change significantly during this transition. The locations of active and inactive alleles based on DNA and RNA FISH signals were also calculated. Inactive *OCT4* alleles were located in very close proximity to the nuclear membrane, whereas active *OCT4* alleles were more centrally disposed in the nucleus. Nevertheless, the nuclear location of active and inactive *SOX2* alleles did not change in either 4‐ or 8‐cell blastomeres. Our RNA and DNA FISH data provide novel information on the allelic expression patterns and positioning of pluripotency‐associated genes, *OCT4* and *SOX2*, during embryonic genome activation in pigs.

## 1 INTRODUCTION

In eukaryotic cells, DNA is contained in the nucleus with heterochromatin concentrated at the periphery and around the nucleolus, and gene‐rich regions are preferentially located in the interior of the nucleus (Cremer & Cremer, 2006; Gilbert, Gilchrist, & Bickmore, 2004; Misteli, 2007). Heterochromatin is a tightly packed form of chromatin and is gene‐poor, possessing markers of closed chromatin such as di‐ and tri‐methylated H3K9 or H3K27 (Guelen et al., 2008). It has been suggested that positioning within the nucleus can affect the expression of heterochromatic regions (Jachowicz, Santenard, Bender, Muller, & Torres‐Padilla, 2013). Several studies have also reported that some genes, but not all, undergo repositioning when gene activity changes (reviewed in Takizawa, Meaburn, & Misteli, 2008). Such genes are those whose activity is tightly linked to differentiation and developmental events, such as *IgH* and *β*‐*globin*, which are activated during the differentiation of B cells and erythroid cells, respectively (Kosak et al., 2002; Ragoczy, Bender, Telling, Byron, & Groudine, 2006), and genes of the hoxB cluster that are involved in embryonic development (Chambeyron & Bickmore, 2004). Similarly, *NANOG* is located at a more internal position within the nucleus of human embryonic stem (ES) cells, where it shows high expression in comparison to its state within the nucleus of differentiated lymphoblastoid cells (Wiblin, 2005). Likewise, *OCT4* shifts its localization from the interior to the surface of its chromosome territory in lymphoblastoid cells (Wiblin, 2005). These observations suggest that a large number of genes likely undergo repositioning during early embryonic development, especially during genome activation when the expression of many genes is significantly up‐ or downregulated. In fact, using DamID technology, Borsos et al. (2019) have observed nuclear reorganization in mouse embryos and this process is not inherited from the maternal germline but is established de novo shortly after fertilization. The two parental genomes establish lamina‐associated domains (LADs) with different features that converge after the 8‐cell stage (Borsos et al., 2019). Positional changes in specific genes during embryonic genome activation in other animals are not well understood.

Maintenance of pluripotency in stem cells or early embryos requires tight regulation of key pluripotency‐associated genes, and one possible mechanism for this may be mono‐allelic expression (Miyanari & Torres‐Padilla, 2012). Studies of mouse ES cells have revealed that *Nanog* expression is subject to mono‐allelic firing (Chambers et al., 2007), possibly through transcriptional bursts (Filipczyk et al., 2013; Hansen & van Oudenaarden, 2013; Navarro et al., 2012). Research on mouse embryos has suggested that the transcriptional activity of *Nanog* is also subject to mono‐allelic bursting and that this is crucial for maintenance of pluripotency, embryonic development, and reprogramming (Miyanari & Torres‐Padilla, 2012). However, little is known about the allelic expression pattern of pluripotency‐associated genes in other mammalian embryos. A better understanding of allelic expression patterns, and the location and repositioning of such important genes, would be potentially useful for embryonic development and stem cell research in pigs. In the present study, we examined the allelic expression pattern and repositioning of the pluripotency‐associated genes, *SOX2* and *OCT4*, in 4‐ and 8‐cell embryos during embryonic genome activation in pigs.

## 2 MATERIALS AND METHODS

### 2.1 Oocyte collection and in vitro maturation (IVM)

Ovaries were collected from prepubertal cross‐bred gilts (Landrace × Large White × Duroc) at a local slaughterhouse and carried to the laboratory in Dulbecco';s phosphate‐buffered saline (PBS) (Nissui Pharmaceutical Co. Ltd.) at 35–37ºC within 1 hr. Cumulus–oocyte complexes (COCs) were collected from follicles 3–6 mm in diameter in collection medium consisting of Medium 199 (with Hanks’ salts; Sigma‐Aldrich Co.) supplemented with 10% fetal bovine serum (Gibco; Thermo Fisher Scientific), 20 mM HEPES (Dojindo Laboratories, Kumamoto, Japan), and antibiotics (100 units/ml penicillin G potassium (Sigma‐Aldrich) and 0.1 mg/ml streptomycin sulfate (Sigma‐Aldrich)). IVM of oocytes was carried out as reported previously (Kikuchi et al., 2002). In brief, about 50 COCs were cultured in 500 µl of maturation medium, which was a modified form of North Carolina State University (NCSU)‐37 solution (Petters & Wells, 1993) containing 10% (v/v) porcine follicular fluid, 0.6 mM cysteine (Sigma‐Aldrich), 50 mM β‐mercaptoethanol (Axon Medchem, Groningen, Netherlands), 1 mM dibutyryl cyclic adenosine 3ʹ,5ʹ‐monophosphate (dbcAMP; Sigma‐Aldrich), 10 IU/ml equine chorionic gonadotropin (Setrotropin; ASKA Pharmaceutical Co. Ltd.), and 10 IU/ml human chorionic gonadotropin (Gonatropin; ASKA) in four‐well dishes (Nunclon Multidishes, Nunc, Thermo Fisher Scientific) for 22 hr in an atmosphere of 5% CO_2_, 5% O_2_, and 90% N_2_ at 38.5ºC. The COCs were subsequently cultured in maturation medium without dbcAMP and hormones for an additional 24 hr.

### 2.2 In vitro fertilization (IVF) and in vitro culture (IVC)

Oocytes were in vitro fertilized according to the two‐step IVF method of Grupen and Nottle (2000) with modifications. The medium used for IVF was a modified Pig‐FM medium (Suzuki et al., 2002) containing 10 mM HEPES, 2 mM caffeine, and 5 mg/ml BSA. The oocytes were washed 3 times in IVF medium, and then transferred into 90‐µl IVF droplets (each containing approximately 20 oocytes) covered with paraffin oil (Paraffin Liquid; Nacalai Tesque, Kyoto, Japan). Frozen‐thawed epididymal spermatozoa from a Meishan boar were preincubated at 38.5°C in Medium 199 (with Earle';s salts, Gibco, Thermo Fisher Scientific, pH adjusted to 7.8) for 15 min (Kikuchi et al., 1998). To obtain the final sperm concentration (1 × 10^5^ sperms/ml), 10 µl of the sperm suspension was introduced into the IVF medium containing oocytes and coincubated for 30 min at 38.5°C under 5% CO_2_, 5% O_2_, and 90% N_2_. The oocytes with the zona‐bound sperms were then transferred to other fresh droplets of the IVF medium and subsequently incubated for 2.5 hr. At the end of IVF, spermatozoa were removed from the surface of the zona pellucida by gentle pipetting with a fine glass pipette. The day of IVF was defined as Day 0.

Presumptive IVF zygotes were then transferred to IVC media. Two types of IVC media were prepared (Kikuchi et al., 2002). The basic IVC medium was NCSU‐37 modified by addition of 0.4% (w/v) BSA and 50 μM β‐mercaptoethanol. Embryos were cultured at 38.5°C under 5% CO_2_, 5% O_2_, and 90% N_2_ in IVC‐PyrLac (basic IVC medium with addition of 0.17 mM sodium pyruvate and 2.73 mM sodium lactate) from Day 0 to Day 2, and then in IVC‐Glu (basic medium supplemented instead with 5.55 mM glucose (Wako Pure Chemical Industries, Ltd., Osaka, Japan)) from Day 2 until collected for RNA and DNA FISH.

### 2.3 RNA and DNA FISH

Four‐ and 8‐cell embryos were collected at 48 and 79 hr after IVF, when developmental ability is known to be high (Nguyen et al., 2020). The zona pellucida was removed by incubation with 1% pronase (protease, Sigma‐Aldrich) for approximately 1 min following by gentle pipetting until the zona pellucida had mostly dissolved, and then the embryos were immediately placed in IVC medium for 5 min. The embryos were washed twice with PBS before being used for RNA and DNA FISH, which were performed according to Miyanari and Torres‐Padilla (2012) with modifications for pig embryos. Briefly, for RNA FISH, selected 4‐ and 8‐cell embryos were incubated in fixative containing 4% paraformaldehyde (Nacalai Tesque) in PBS for 20 min at room temperature. The embryos were permeabilized in fixative for 10 min at room temperature. After washing with PBS three times, the embryos were prehybridized in a 4 μl drop of hybridization buffer covered with mineral oil for 30 min at 50°C. The hybridization buffer consisted of 50% formamide (Wako), 10% dextran sulfate (Wako), 2x SSC, 1 μg/μl Hybloc DNA (Applied Genetics Laboratories), 1 mM vanadyl ribonucleotide complex (Sigma‐Aldrich), 1 mg/ml polyvinyl pyrrolidone (PVP), 0.05% Triton X‐100, and 0.5 mg/ml BSA. The embryos were then transferred to a 4 μl drop of hybridization buffer containing 10 ng/μl ChromaTide Alexa Fluor 488 fluorescent probe and incubated at 50°C overnight. After three washes with 2x SSC solution supplemented with 0.1% Triton X‐100 and 1 mg/ml PVP at 50°C for 10 min, the embryos were mounted on glass slides and stained with Vectashield containing DAPI. Images were acquired on a LSM700 laser scanning microscope (Carl Zeiss, Oberkochen, Germany) with a 488 oil immersion objective lens.

For DNA FISH, selected 4‐ and 8‐cell embryos were incubated in fixative containing 4% paraformaldehyde in PBS for 15 min at room temperature, and then permeabilized in fixative for 1 hr at 37°C. RNase was also added to the permeabilization buffer to digest the RNA. After briefly washing with PBS, the embryos were briefly treated with 0.3% HCl solution at room temperature to remove histones. After two washes with PBS, the embryos were equilibrated in a 4‐μl drop of hybridization buffer covered with mineral oil for 3 hr at 55°C. The hybridization buffer consisted of 50% formamide, 10% dextran sulfate, 2x SSC, 1 µg/μl Hybloc DNA, 5 mM EDTA, 1 mg/ml PVP, 0.1% Triton X‐100, and 1 mg/ml BSA. Denaturation was performed by incubation at 83°C for 10 min. The embryos were then prehybridized for 1 hr at 37°C before being transferred to a 4‐μl drop of hybridization buffer containing 10 ng/μl fluorescent probe and incubated at 37°C overnight. After a brief wash with 2× SSC and twice with 0.2× SSC solution supplemented with 0.1% Triton X‐100 and 1 mg/ml PVP at 52°C for 15 min, the embryos were mounted on glass slides and stained with Vectashield containing DAPI. Images were acquired on a laser scanning microscope with a 488‐oil immersion objective lens.

Coding regions of *SOX2, OCT4, TUBA,* and *ACTB* were amplified using ExTaq DNA Polymerase (TaKaRa) with the specific primers (Table 1). The PCR products were confirmed by DNA sequencing using an Applied Biosystems 3130xl Genetic Analyzer and then used as probes for RNA and DNA FISH. Probes were labeled with ChromaTide Alexa Fluor 488‐dATP using Degenerate Oligonucleotide Primed (DOP) PCR according to Backx, Thoelen, Van Esch, and Vermeesch (2008) and purified with a QIAquick PCR Purification Kit (QIAGEN).

**Table 1:** Primer sequences used for probe production

Gene symbol	Ensembl ID	Primer sequences
*SOX2*	ENSSSCG00000011771	Forward 5’‐AGGATCGGCCAGAAGAGG−3’ Reverse 5’‐AAGCGTACCGGGTTTTTCTC−3’
*OCT4*	ENSSSCG00000001393	Forward 5’‐CTTGGAGAGCCCTGGTTTTA−3’ Reverse 5’‐GATGTCCTGGGACTGCATTT−3’
*ACTB*	ENSSSCG00000007585	Forward 5’‐ACACGGAGTACTTGCGCTCT−3’ Reverse 5’‐GACCAGCGTTTGCCTTTTAT−3’
*TUBA*	ENSSSCG00025075974	Forward 5’‐ACGGTAACTCCTCCCGTTCT−3’ Reverse 5’‐GAGACGCCTTGACTCTGGAC−3’

### 2.4 Statistical analysis

**Figure 1:**
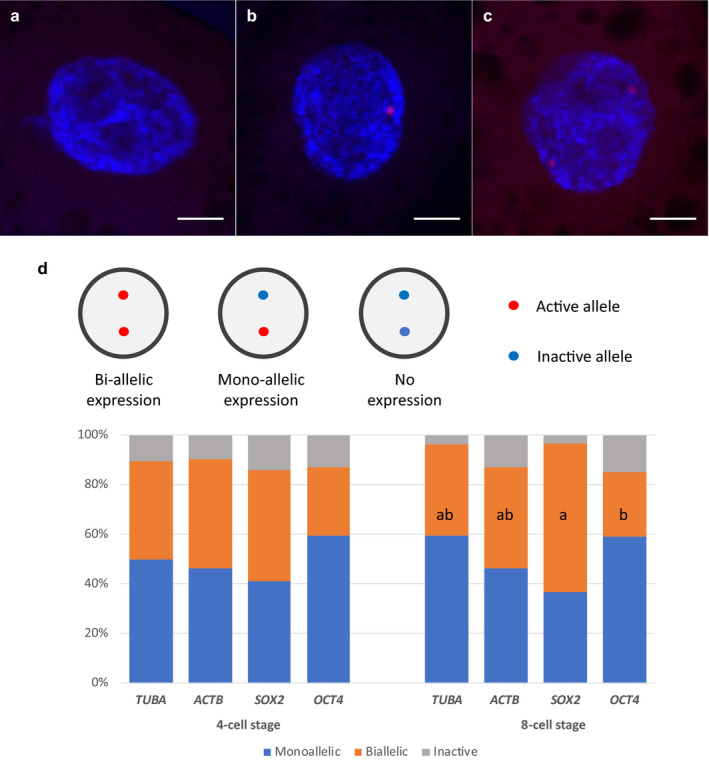
Allelic expression pattern of selected genes in porcine embryos. A 4‐cell stage blastomere with no (a), mono‐allelic (b), and bi‐allelic expression (c) for *SOX2*. Allelic expression pattern for selected pluripotency‐associated and house‐keeping genes in 4‐ and 8‐cell stage in porcine embryos (d). Number of blastomeres assessed for a specific gene at a specific stage ranged from 30 to 101. Scale bar presents 5 μm. Letters (a,b) denote significant difference (*p* < .05)

**Figure 2:**
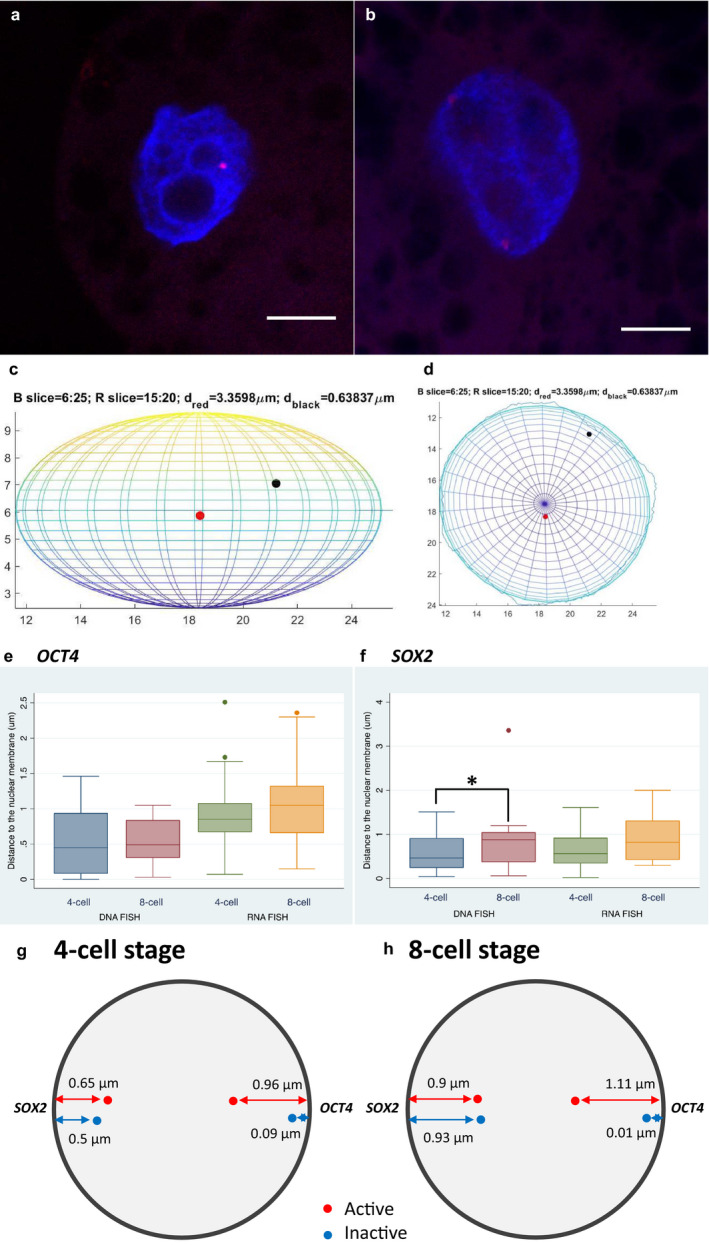
Nuclear location of pluripotency‐associated genes in 4‐ and 8‐cell stages in porcine embryos. An RNA FISH image of an 8‐cell stage blastomere (a) and a DNA image of a 4‐cell stage blastomere (b). Construction of nuclear territory and identification of RNA/DNA FISH signal in z (c) and in xy (d) using MATLAB. Location of *OCT4* (e) and *SOX2* (f) alleles, determined by distance of the detected FISH signals to the nuclear membrane, in 4‐ and 8‐cell embryos by RNA and DNA FISH techniques. Nuclear location of active and inactive *OCT4* and *SOX2* alleles in 4‐ (g) and 8‐cell embryos (h) calculated based on RNA (active alleles) and DNA FISH signals (both active and inactive alleles). Number of blastomeres assessed for a specific gene at a specific stage ranged from 16 to 56. Scale bar presents 5 μm. ^*^ *p* = .089

Only interphase nuclei were selected for image analysis, which was performed using MATLAB version 2018 and ImageJ. Blastomere nuclear territory and positions of RNA/DNA signals in the nucleus were calculated in three dimensions by a MATLAB script using the fit_ellipse.m function written by Gal (2019) [https://www.mathworks.com/matlabcentral/fileexchange/3215‐fit_ellipse]. Detected fluorescent spots located inside nuclear territory that were distinguishable from the background were considered real signals. Blastomeres with no, one, or two well‐separated RNA FISH signals were scored as no expression, mono‐allelic, or bi‐allelic expression (Figure 1a‐c, Figure 2a). Only nuclei with two DNA FISH signals were used for location calculation (Figure 2b). Two fluorescent spots in very close proximity, which might have resulted from DNA replication, were recorded as one signal. The position of a signal was the nearest distance from its center of mass to the nuclear membrane. Embryos in which all blastomeres showed no fluorescent signals were excluded from further image analysis.

All data were expressed as mean ± *SD* values. The data were analyzed by one‐way ANOVA followed by Bonferroni correction by using the Stata/SE 15.0 software package (StataCorp., College Station, TX, USA). Differences at *p* < .05 were considered to be statistically significant.

## 3 RESULTS

### 3.1 Allelic gene expression during early development

The allelic expression patterns of two pluripotency‐associated genes, *OCT4* and *SOX2*, and two housekeeping genes, *ACTB* and *TUBA*, were examined in 4‐ and 8‐cell embryos. The proportions of blastomeres with mono‐, bi‐allelic, or no expression for *OCT4* in 4‐cell embryos were similar to those in 8‐cell embryos (Figure 1d). Likewise, no significant changes in *TUBA* and *ACTB* allelic expression patterns were recorded during 4‐ to 8‐cell stage transition. Although it is not significant, the proportion of blastomeres bi‐allelically expressing *SOX2* increased from 45% in 4‐cell embryos to 60% in 8‐cell embryos (Figure 1d).

The ratios of mono‐, bi‐allelic, and no expression for all four investigated genes were similar at the 4‐cell stage. However, in 8‐cell embryos, the percentage of blastomeres that expressed *SOX2* bi‐allelically was significantly higher than was the case for *OCT4* (Figure 1d).

### 3.2 Gene repositioning during early development

The radial location of all *OCT4* alleles and active *OCT4* alleles in the nucleus, determined on the basis of DNA and RNA FISH signals, did not change significantly during 4‐ to 8‐cell transition (Figure 2e). Similarly, the distance from active *SOX2* alleles to the nuclear membrane was comparable at these developmental stages. However, *SOX2* alleles tended to move toward the nuclear interior during 4‐ to 8‐cell transition (*p* = .089) (Figure 2f).

Based on DNA and RNA FISH signals, the locations of active and inactive alleles were calculated (Figure 2g, h). The distance to the nuclear membrane from active and inactive *SOX2* alleles did not differ significantly in both 4‐ and 8‐cell blastomeres. Inactive *OCT4* alleles were located in very close proximity to the nuclear membrane, whereas active *OCT4* alleles were more centrally disposed in the nucleus during 4‐ to 8‐cell transition (Figure 2g, h).

## 4 DISCUSSION

The allelic expression pattern and repositioning of several genes, including pluripotency‐associated genes, has been reported in human somatic and stem cells (Croft et al., 1999; Wiblin, 2005), mouse somatic cells, stem cells, and embryonic cells during differentiation and early embryonic development (Borsos et al., 2019; Chambeyron & Bickmore, 2004; Kosak et al., 2002; Miyanari & Torres‐Padilla, 2012; Ragoczy et al., 2006). However, such information for any animal other than mice has not been published so far. In the present study, we examined the allelic expression pattern and nuclear location of two pluripotency‐associated genes, *OCT4* and *SOX2*, during embryonic genome activation in pigs. The location of these genes in the nucleus was determined in terms of the radial distance from the gene alleles to the nuclear membrane. We also have tried to examine allelic expression of *NANOG* which is another pluripotency‐associated gene. However, *NANOG* expression could not be detected by FISH according to technical problems.

We did not detect any striking differences in the allelic expression patterns of *OCT4* and *SOX2*, or the two housekeeping genes, *ACTB* and *TUBA*, in both 4‐ and 8‐cell embryos. The proportions of blastomeres with mono‐, bi‐allelic, or no expression for all four of these genes were similar at both the 4‐ and 8‐cell developmental stages (Figure 1d). Notably, the percentages of mono‐allelic expression were comparable to those of bi‐allelic expression for all four examined genes and in both stages. Likewise, no significant changes in the allelic expression pattern of *SOX2, OCT4*, *TUBA,* and *ACTB* were recorded during 4‐ to 8‐cell stage transition. In mouse embryos, it has been reported that – except for *Nanog* – the majority of blastomeres show bi‐allelic expression for all of the examined genes, including *Sox2, Oct4,* and *Actb* (Miyanari & Torres‐Padilla, 2012). These data suggest that there are differences in allelic expression profiles between pig and mouse embryos during early embryonic development. However, it should be noted that in the study by Miyanari and Torres‐Padilla (2012), cells with two, three, or four separate RNA‐FISH spots were scored as bi‐allelism, whereas in our present study only blastomeres with two RNA‐FISH spots were defined as showing bi‐allelic expression. This difference in the definition of bi‐allelism might have partly contributed to the contrasting results between the two studies. Another factor that might also contribute to the difference between mouse and pig embryos is the synchronization of blastomere division. Using time‐lapse recording, it has been observed that, in pigs, the timing of division of some blastomeres may lag hours behind others (Anderson, Matteri, Abeydeera, Day, & Prather, 1999; Mateusen et al., 2005). In our preliminary experiments to optimize the timing of collection of 4‐ and 8‐cell embryos (Nguyen et al., 2020), we usually found higher numbers of 5‐ to 7‐cell embryos than 4‐ and 8‐cell embryos at any time point of assessment. Furthermore, many 3‐cell embryos were also found. In contrast, blastomeres in mouse embryos show somewhat synchronized division, with differences of only a few dozen minutes. These findings suggest that some of the blastomeres in the same embryos used in our study were arrested in different phases of the cell cycle. It has been reported that the gene expression profile of cells depends on the cell cycle phases (Grant et al., 2013; Liu et al., 2017). Although no report has documented this issue, cell cycle phase might also affect the allelic expression of certain genes. Utilization of inhibitors to synchronize the cell cycle transition in blastomeres is one potential solution to this problem. However, since most inhibitors interfere with embryonic development, we opted for natural development. Another point to note is that the use of RNA FISH only yields allelic expression data at the point of fixation, and is unable to indicate whether there are transcriptional bursts, where a gene allele switches between active and inactive states, or random allelic fluctuations, where the two alleles of a gene show ordered activation. In order to catch such events, live imaging is required. However, for porcine embryos, live imaging is rather challenging due to the large amount of lipid droplets stored in the blastomeres.

Interestingly, we found that—although not significant—the proportion of blastomeres bi‐allelically expressing *SOX2* increased from 45% in 4‐cell embryos to 60% in 8‐cell embryos, and that *SOX2* was expressed bi‐allelically in a significantly greater number of blastomeres than was the case for *OCT4* in 8‐cell embryos (Figure 1d). This corresponded to our finding that *SOX2* alleles tended to move toward the nuclear interior during 4‐ to 8‐cell transition (Figure 2f), whereas the location of *OCT4* alleles in the nucleus did not change significantly (Figure 2e). Taken together, these observations may indicate a correlation between a change in the allelic expression pattern of *SOX2* and its repositioning during early embryonic development in pigs. However, it is noteworthy that one of the examined *SOX2* alleles was located 3.36 μm away from the nuclear membrane, which was considerably further than the remaining one, for which the average distance was 0.92 μm (Figure 2e).

We also calculated the radial locations of active and inactive alleles based on their DNA and RNA FISH signals. This revealed that inactive *OCT4* alleles were located in very close proximity to the nuclear membrane, whereas active *OCT4* alleles occupied the more inner area of the nucleus (Figure 2g, h). Such behavior of *OCT4* alleles can be considered evidence for a correlation between gene expression and gene positioning. This is in agreement with a report by Stachecka, Nowacka‐Woszuk, Kolodziejski, and Szczerbal (2019), who demonstrated that active *PPARG* alleles are centrally positioned when inactive. Nevertheless, our data also demonstrated that the distance of active and inactive *SOX2* alleles from the nuclear membrane did not differ significantly in both 4‐ and 8‐cell blastomeres. Taken together, these data suggest that although there is evidence to support a correlation between gene expression and gene positioning, this might not apply to all genes during embryonic genome activation in pigs. Therefore, to obtain a better perspective on the correlation between gene expression and gene positioning, more genes should be studied. It should also be noted that, in our study, the nuclear locations of inactive alleles were computed indirectly based on the locations of active alleles and all alleles, as determined by RNA and DNA FISH, respectively. Sequential RNA‐DNA FISH would allow direct measurement, and therefore yield more accurate results. Furthermore, artifacts might also lead to errors in the determination of allele locations using RNA and DNA FISH. One such artifact is the more oval rather than spherical shape of the nucleus, as shown in Figure 2c, despite our efforts to maintain the nuclear shape.

It is well documented that porcine embryos are characterized by nuclear and ploidy abnormalities (Dang‐Nguyen et al., 2010; McCauley et al., 2003; Ulloa Ulloa et al., 2008), which would affect the results of FISH. In order to eliminate such aberrations, we carefully optimized methods for minimizing these abnormalities in the embryos we used for the FISH assays, which included strict selection of oocytes for IVF and embryos following IVF based on their morphology and the timing of early cleavages. This approach for optimizing the production and selection of good embryos has been proven to significantly reduce the incidence of nuclear and ploidy abnormalities (Nguyen et al., 2020).

In summary, our data obtained using RNA and DNA FISH have yielded novel information on allelic expression patterns and positioning of two pluripotency‐associated genes, *OCT4* and *SOX2*, during embryonic genome activation in pigs. We have shown that the repositioning of *SOX2* alleles coincided with an increase in the percentage of blastomeres with bi‐allelic expression during these stages of embryonic development, and that there was a correlation between the expression and nuclear location of *OCT4*. This information should be useful for improvement of embryonic development and stem cell research.

## CONFLICT OF INTEREST

All authors declare no conflict of interest.
